# Effect of Cervical Restorations on Periodontal Health: An Observational Cross-Sectional Study

**DOI:** 10.7759/cureus.110471

**Published:** 2026-06-08

**Authors:** Mohd Zeeshan Ahmad, Nitish Mittal, Shreya Bhukal, Khushabu Deshmukh, Sachin Sarjerao Deshmukh, Banashree Sankeshwari, Priyanka Vhanmane

**Affiliations:** 1 Department of Conservative Dentistry and Endodontics, Rama Dental College Hospital and Research Centre, Kanpur, IND; 2 Department of Conservative Dentistry and Endodontics, Desh Bhagat Dental College and Hospital, Mandi Gobindgarh, IND; 3 Department of Cardiology, All India Institute of Medical Sciences, New Delhi, IND; 4 Department of Periodontology, Saraswati Dhanwantari Dental College and Hospital, Parbhani, IND; 5 Department of Prosthodontics, Bharati Vidyapeeth (Deemed to be University) Dental College and Hospital, Sangli, IND; 6 Department of Periodontology, Bharati Vidyapeeth (Deemed to be University) Dental College and Hospital, Sangli, IND

**Keywords:** cervical restorations, composite resin, glass ionomer cement, periodontal health, restoration margins

## Abstract

Introduction

Cervical restorations are commonly used to manage cervical lesions and defects involving the tooth structure near the gingival margin. Understanding the periodontal response associated with cervical restorations is essential for improving the long-term restorative success and periodontal stability. The present study aimed to evaluate the effect of cervical restorations on periodontal health and assess the influence of restoration margin position and restorative material type on periodontal parameters.

Materials and methods

This observational cross-sectional, split-mouth study included 120 teeth in 60 patients, divided into cervical restoration group (n = 60) and contralateral healthy teeth as control (n = 60) group. The periodontal parameters assessed included plaque index (PI), gingival index (GI), probing pocket depth (PPD), and clinical attachment level (CAL). Restorations were further categorized according to margin location as supragingival, equigingival, or subgingival and according to restorative materials such as composite resin, glass ionomer cement (GIC), and resin-modified GIC (RMGIC). Statistical analyses were performed using the Mann-Whitney U test, Kruskal-Wallis test, and Dunn-Bonferroni post hoc analysis, with p < 0.05 considered statistically significant.

Results

The restored group demonstrated significantly higher PI, GI, PPD, and CAL values than did the control group (p < 0.001). Subgingival restorations exhibited the highest periodontal parameter values, whereas supragingival restorations exhibited the lowest values (p < 0.001). Composite resin restorations demonstrated significantly poorer periodontal outcomes than GIC and RMGIC restorations (p < 0.05). No significant differences were observed between GIC and RMGIC restorations (p > 0.05).

Conclusions

Cervical restorations are associated with poorer periodontal health, particularly when restoration margins are placed subgingivally. Composite resin restorations have demonstrated less favorable periodontal outcomes than glass ionomer-based materials. Appropriate restorative material selection, careful margin placement, and regular periodontal maintenance are important for preserving periodontal health around cervical restorations.

## Introduction

Cervical restorations are routinely used in clinical dental practice to restore non-carious cervical lesions, root caries, abrasion, erosion, and abfraction defects. Despite their widespread use and restorative benefits, concerns remain regarding their influence on adjacent periodontal tissues, particularly when restoration margins are placed close to or below the gingival margin [1,2]. Improper contours, marginal overhangs, surface roughness, and plaque-retentive restorative materials may contribute to biofilm accumulation, gingival inflammation, periodontal pocket formation, and attachment loss. The long-term success of cervical restorations depends not only on restorative durability and esthetics but also on the maintenance of periodontal health [2,3]. Recent studies have emphasized the importance of restorative material selection and long-term restorative performance in maintaining oral health and treatment success [4].

The relationship between restorative procedures and periodontal health has been extensively discussed in the literature. Violation of the biological width, subgingival margin placement, and inadequate finishing or polishing of restorations have been identified as important factors associated with adverse periodontal responses [5,6]. Different restorative materials, such as composite resin, glass ionomer cement (GIC), and resin-modified GIC (RMGIC), exhibit varying surface characteristics, fluoride-release properties, and plaque-retentive tendencies, which may influence periodontal outcomes differently [7,8]. Furthermore, the location of restoration margins relative to gingival tissues may significantly affect plaque accumulation and gingival inflammation [1]. Although previous studies have evaluated restorative margins and periodontal parameters, evidence comparing the periodontal effects of various cervical restorative materials and margin positions remains limited and inconsistent [9,10].

Understanding the periodontal implications of cervical restorations is clinically important for selecting appropriate restorative materials and techniques to minimize periodontal complications [1,2]. Therefore, the present observational cross-sectional study was designed to evaluate the effect of cervical restorations on periodontal health by comparing the periodontal parameters between restored and non-restored teeth. This study also aimed to assess the influence of restoration margin location on periodontal status and to compare periodontal outcomes among different cervical restorative materials, including composite resin, GIC, and RMGIC.

## Materials and methods

Study design and setting

This observational cross-sectional, split-mouth study was conducted in the Department of Periodontology in collaboration with the Department of Conservative Dentistry and Endodontics at Bharati Vidyapeeth (Deemed to be University) Dental College and Hospital, Sangli, India, from March 2024 to December 2024. The study protocol was reviewed and approved by the Institutional Ethics Committee (approval BVDU/DCH//03/2023-24) prior to commencement, and all procedures were performed in accordance with the ethical principles outlined in the Declaration of Helsinki. Written informed consent was obtained from all participants prior to their enrollment in the study. No restorative or periodontal intervention was performed as part of the study protocol, and the investigators only recorded existing clinical conditions at the time of examination.

Study population

Patients reporting to the outpatient departments of periodontology and conservative dentistry during the study period were screened for eligibility. Consecutive eligible patients presenting during the study period were recruited. The inclusion and exclusion criteria are presented in Table 1.

**Table 1:** Inclusion and exclusion criteria for study participants

Inclusion criteria	
1	Patients aged between 20 and 45 years
2	Presence of at least one tooth with a cervical restoration extending to or near the gingival margin
3	Cervical restoration present for a minimum duration of six months
4	Presence of a minimum of 20 natural teeth
5	Willingness to provide informed consent
Exclusion criteria	
1	Patients with systemic diseases known to influence periodontal health (such as uncontrolled diabetes mellitus or immunocompromised conditions)
2	Smokers or tobacco users
3	Pregnant or lactating women
4	Patients undergoing orthodontic treatment
5	Patients who had received periodontal therapy within the preceding six months
6	Patients who had received antibiotic therapy within the preceding six months
7	Teeth with crowns or fixed partial dentures
8	Teeth with extensive prosthetic restorations involving the cervical area
9	Teeth with mobility greater than Grade II

Sample size estimation

Sample size estimation was performed using the G*Power software version 3.1 (Heinrich Heine University, Düsseldorf, Germany). Based on an effect size of 0.58 obtained from a previous study evaluating the effects of cervical restorations on periodontal tissues, with a confidence level of 95%, statistical power of 80%, and alpha error of 0.05, the minimum required sample size was calculated as 48 teeth per group for an independent group comparison [10]. To compensate for possible dropouts and to facilitate subgroup analyses based on restorative materials and margin locations, 60 teeth were included in each group, resulting in a total sample size of 120 teeth in 60 patients.

Grouping of study samples

The study teeth were classified into two observational categories. Group I consisted of teeth with cervical restorations, while, whenever feasible, contralateral or adjacent non-restored teeth from the same participant were selected as controls (Group II) to minimize inter-individual variability. The cervical restorations evaluated in this study included composite resin, GIC, and RMGIC. Restorations were further classified according to the location of the restoration margin relative to the gingival margin as supragingival, equigingival, or subgingival [5].

Clinical examination procedure

All clinical examinations were performed by a single calibrated examiner under standardized illumination using a mouth mirror (Hu-Friedy Manufacturing Co., Chicago, Illinois, USA) and a UNC-15 periodontal probe (Hu-Friedy Manufacturing Co.). Prior to the commencement of the study, intra-examiner calibration was performed on 10 patients examined twice at a one-week interval, and a kappa coefficient greater than 0.80 was considered acceptable for reproducibility. Demographic variables, including age, sex, oral hygiene practices, brushing frequency, type of restoration, and duration of restoration, were recorded. Periodontal parameters were assessed at six sites per tooth and included plaque index (PI), gingival index (GI), probing pocket depth (PPD), and clinical attachment level (CAL), all measured in millimeters [11]. The examiner recording periodontal parameters was blinded to the type of restorative material during clinical assessment whenever feasible.

Outcome measures

The primary outcome measure of this study was the association between cervical restoration and periodontal health parameters. Secondary outcome measures included the influence of restoration margin location on periodontal status and comparison of periodontal parameters among different restorative materials.

Statistical analysis

The data obtained from the study were statistically analyzed using IBM SPSS Statistics for Windows, version 25.0 (released 2017; IBM Corp., Armonk, NY, USA). Descriptive statistics were expressed as medians and IQRs for continuous variables and frequencies with percentages for categorical variables. Normality of data distribution was assessed using the Shapiro-Wilk test, which demonstrated a non-normal distribution of the study variables. Accordingly, comparisons between the restored and control groups were performed using the Mann-Whitney U test. Comparisons among multiple subgroups based on restorative materials and restoration margin positions were performed using the Kruskal-Wallis test followed by Dunn-Bonferroni post hoc analysis. Statistical significance was set at p < 0.05.

## Results

A total of 120 teeth from 60 participants were included in the study, comprising 60 (50%) in the cervical restoration group and 60 (50%) in the control group. The study participants comprised a near-equal gender distribution, with 32 males (53.3%) and 28 females (46.7%). Regarding age, the majority were in the 30-39 years group, followed by those aged 40-45 years and 20-29 years. In terms of oral hygiene practices, most participants reported brushing twice daily, while 22 (36.7%) brushed once daily, and only four (6.6%) had irregular brushing habits (Table 2).

**Table 2:** Demographic and clinical profile of study participants (N = 60) Data presented as frequency and percentage (%), where n denotes the number of participants.

Demographic variable	Category	n (%)
Sex	Male	32 (53.3)
	Female	28 (46.7)
Age group (years)	20-29	14 (23.3)
	30-39	28 (46.7)
	40-45	18 (30.0)
Brushing frequency	Once daily	22 (36.7)
	Twice daily	34 (56.7)
	Irregular	4 (6.6)

A comparison of periodontal parameters between the study groups demonstrated significantly poorer periodontal health in teeth with cervical restorations (Table 3). The restored group showed significantly higher median PI values than did the control group (p < 0.001). Similarly, the GI values were significantly higher in the restored group than in the control group (p < 0.001). PPD and CAL were also significantly higher in restored teeth, with median values of 3.20 mm (2.80-3.70) and 2.90 mm (2.40-3.40), respectively, compared to 2.10 mm (1.80-2.50) and 1.80 mm (1.50-2.20) in the control group (p < 0.001 for both).

**Table 3:** Comparison of periodontal parameters between Group I (restored) and Group II (control) * p < 0.05 was considered statistically significant. Group I = teeth with cervical restorations; Group II = non-restored control teeth Data are presented as median (IQR); the Mann-Whitney U test was used for comparison. CAL, clinical attachment level; GI, gingival index; PI, plaque index; PPD, probing pocket depth

Parameter	Group I: Cervical restoration (median (IQR), n = 60)	Group II: Control (median (IQR), n = 60)	U statistic	Degree of freedom	p-value	Effect size
PI	1.80 (1.50-2.10)	1.20 (1.00-1.50)	812.5	118	<0.001*	1.27
GI	1.70 (1.40-2.00)	1.10 (0.90-1.40)	785	118	<0.001*	1.31
PPD (mm)	3.20 (2.80-3.70)	2.10 (1.80-2.50)	702	118	<0.001*	1.44
CAL (mm)	2.90 (2.40-3.40)	1.80 (1.50-2.20)	668.5	118	<0.001*	1.48

Analysis of the restoration margin position revealed a progressive increase in periodontal parameter values from the supragingival to equigingival and subgingival restorations (Table 4). The teeth with subgingival restoration margins exhibited the highest median PI, GI, PPD, and CAL values. In contrast, supragingival restorations demonstrated the lowest periodontal parameter values. Kruskal-Wallis analysis revealed statistically significant differences among the three margin positions for all periodontal parameters (p < 0.001).

**Table 4:** Influence of cervical restoration margin position on periodontal parameters * p < 0.05 was considered statistically significant. Data are presented as median (IQR); the Kruskal-Wallis test was used for comparison. CAL, clinical attachment level; GI, gingival index; PI, plaque index; PPD, probing pocket depth

Parameter	Supragingival (n = 20), median (IQR)	Equigingival (n = 20), median (IQR)	Subgingival (n = 20), median (IQR)	Kruskal-Wallis H statistic	Degree of freedom	p-value	Effect size
PI	1.40 (1.20-1.70)	1.80 (1.50-2.10)	2.20 (1.90-2.50)	18.42	2	<0.001*	0.29
GI	1.30 (1.10-1.60)	1.70 (1.40-2.00)	2.10 (1.80-2.40)	20.15	2	<0.001*	0.32
PPD (mm)	2.50 (2.20-2.90)	3.10 (2.70-3.50)	3.80 (3.40-4.20)	22.63	2	<0.001*	0.36
CAL (mm)	2.20 (1.90-2.60)	2.80 (2.40-3.20)	3.50 (3.10-3.90)	24.48	2	<0.001*	0.39

Post hoc Dunn-Bonferroni analysis demonstrated significant pairwise differences between the supragingival, equigingival, and subgingival margin positions for PI, GI, PPD, and CAL (Table 5). The greatest differences were observed between the supragingival and subgingival restorations, with all comparisons showing highly significant p-values. Equigingival restorations also demonstrated significantly poorer periodontal parameters than supragingival restorations, whereas subgingival restorations exhibited significantly worse outcomes than equigingival restorations.

**Table 5:** Post hoc Dunn-Bonferroni analysis for restoration margin position * p < 0.05 was considered statistically significant. Data are presented as Z values and p-values; the Dunn-Bonferroni post hoc test was used for pairwise comparison. CAL, clinical attachment level; GI, gingival index; PI, plaque index; PPD, probing pocket depth

Comparison	PI (Z value)	p-value	GI (Z value)	p-value	PPD (Z value)	p-value	CAL (Z value)	p-value
Supragingival vs. equigingival	-2.31	0.041*	-2.38	0.038*	-2.52	0.029*	-2.67	0.021*
Supragingival vs. subgingival	-4.86	<0.001*	-5.12	<0.001*	-5.48	<0.001*	-5.76	<0.001*
Equigingival vs. subgingival	-2.44	0.033*	-2.58	0.027*	-2.73	0.018*	-2.81	0.015*

A comparison of the different restorative materials demonstrated significant variations in periodontal outcomes (Table 6). Composite resin restorations exhibited the highest median PI, GI, PPD, and CAL values, indicating poorer periodontal status than GIC and RMGIC restorations. GIC restorations showed the lowest periodontal parameter values, while RMGIC restorations demonstrated intermediate findings. Statistically significant differences were observed between the restorative materials for all periodontal parameters (p < 0.05).

**Table 6:** Comparison of periodontal parameters across restoration material types * p < 0.05 was considered statistically significant. Data are presented as median (IQR); the Kruskal-Wallis test was used for comparison. CAL, clinical attachment level; GI, gingival index; GIC, glass ionomer cement; PI, plaque index; PPD, probing pocket depth; RMGIC, resin-modified glass ionomer cement

Parameter	GIC (n = 20), median (IQR)	Composite (n = 20), median (IQR)	RMGIC (n = 20), median (IQR)	Kruskal-Wallis H statistic	Degree of freedom	p-value	Effect size
PI	1.60 (1.30-1.90)	2.10 (1.80-2.40)	1.70 (1.40-2.00)	11.86	2	0.003*	0.17
GI	1.50 (1.20-1.80)	2.00 (1.70-2.30)	1.60 (1.30-1.90)	13.22	2	0.001*	0.2
PPD (mm)	2.80 (2.40-3.20)	3.60 (3.20-4.00)	3.00 (2.60-3.40)	15.34	2	<0.001*	0.23
CAL (mm)	2.50 (2.10-2.90)	3.30 (2.90-3.70)	2.70 (2.30-3.10)	16.07	2	<0.001*	0.25

Post hoc analysis revealed that composite restorations were associated with significantly poorer periodontal outcomes than both GIC and RMGIC restorations (Table 7). Comparisons between GIC and composite restorations showed statistically significant differences in all periodontal parameters. Similarly, composite restorations demonstrated significantly higher periodontal parameter values than RMGIC restorations. However, no statistically significant differences were observed between the GIC and RMGIC restorations for any periodontal parameter (p > 0.05).

**Table 7:** Post hoc Dunn-Bonferroni analysis among restorative materials * p < 0.05 was considered statistically significant. Data are presented as Z values and p-values; the Dunn-Bonferroni post hoc test was used for pairwise comparison. CAL, clinical attachment level; GI, gingival index; GIC, glass ionomer cement; PI, plaque index; PPD, probing pocket depth; RMGIC, resin-modified glass ionomer cement

Comparison	PI (Z value)	p-value	GI (Z value)	p-value	PPD (Z value)	p-value	CAL (Z value)	p-value
GIC vs. Composite	-3.04	0.004*	-3.28	0.002*	-3.86	<0.001*	-4.02	<0.001*
GIC vs. RMGIC	-0.81	0.418	-0.86	0.392	-1.07	0.287	-1.02	0.301
Composite vs. RMGIC	-2.71	0.011*	-2.84	0.009*	-3.02	0.006*	-3.18	0.004*

## Discussion

The findings of the present study demonstrated significantly higher PI, GI, PPD, and CAL values in restored teeth than in non-restored controls, suggesting that cervical restorations may adversely influence periodontal health when plaque-retentive factors are present. The increased periodontal inflammation observed around the restored teeth may be attributed to several restorative and biological factors. Cervical restorations positioned close to the gingival margin can create areas of plaque retention owing to marginal discrepancies, overcontouring, inadequate finishing and polishing, or increased surface roughness [1]. These conditions favor bacterial biofilm accumulation, resulting in persistent gingival inflammation and subsequent periodontal destruction [12]. Similar findings were reported by Favetti et al. [13], who observed that cervical restorations influenced periodontal tissue conditions over a long-term follow-up period. Previous studies have also demonstrated that restoration-associated plaque accumulation contributes to increased gingival bleeding, attachment loss, and periodontal pocket formation, particularly when restorative margins violate the biological width [5,6].

The present study further demonstrated that the restoration margin position significantly affected periodontal outcomes, with subgingival margins exhibiting the highest PI, GI, PPD, and CAL values, whereas supragingival margins showed the most favorable periodontal conditions. The preservation of periodontal health is an essential consideration during restorative procedures involving cervical tooth surfaces. Improperly positioned restorative margins, particularly those extending subgingivally, may violate supracrestal tissue attachment and contribute to plaque accumulation, chronic gingival inflammation, periodontal pocket formation, and attachment loss [12]. Therefore, the biological relationship between restorative margins and periodontal tissues plays a crucial role in determining the long-term success of cervical restorations [14]. Previous literature has emphasized that restoration margins placed too close to the gingival attachment may adversely affect periodontal stability and the maintenance of oral hygiene [12,15]. The greater periodontal destruction associated with subgingival margins may be explained by impaired accessibility for oral hygiene maintenance, increased plaque retention, and a chronic inflammatory response caused by continuous microbial colonization [12].

Another important finding of the present study was the variation in periodontal parameters among different restorative materials. Composite resin restorations demonstrated significantly poorer periodontal outcomes than GIC and RMGIC restorations. This difference may be related to the surface characteristics and plaque-retentive potential of the composite materials. Composite restorations are more susceptible to marginal degradation and increased surface roughness over time, which may facilitate microbial adhesion and biofilm maturation [16]. In contrast, GIC and RMGIC possess fluoride-releasing properties that may inhibit bacterial colonization and reduce plaque accumulation [7,17]. Furthermore, glass ionomer-based materials exhibit better marginal adaptation and improved biocompatibility with gingival tissues, which may explain their comparatively favorable periodontal response [18]. Previous studies have similarly reported lower plaque accumulation, improved gingival health, and better marginal adaptation around the RMGIC and resin-based composite resins [19,20].

The findings of the present study have important clinical implications for restorative and periodontal practice. Careful selection of restorative materials and proper margin placement are essential for maintaining long-term periodontal health. Whenever clinically feasible, supragingival margin placement is preferred to minimize plaque accumulation and facilitate oral hygiene maintenance. Restorative materials with favorable biological and surface properties, such as GIC and RMGIC, may provide better periodontal compatibility in cervical lesions located near the gingival margin. Meticulous finishing and polishing procedures, along with regular periodontal maintenance, are necessary to reduce restoration-associated periodontal complications.

Despite these significant findings, the present study has several limitations. The cross-sectional design precludes the establishment of causal relationships between cervical restorations and periodontal parameters. The study was conducted at a single institution with a relatively limited sample size, which may restrict the generalizability of the findings. Variations in restoration age, operator technique, oral hygiene practices, and restoration quality were not extensively controlled and may have influenced the observed associations. Furthermore, complete examiner blinding was not feasible because restorative materials and margin locations were clinically visible during examination, potentially introducing observer bias. The exclusion of smokers and medically compromised individuals may further limit the external validity of the results. In addition, potential clustering of teeth within individual patients was not specifically accounted for in the statistical analysis and may have affected the precision of the estimates. Radiographic and microbiological assessments were not performed. Future longitudinal, multicenter studies with larger and more diverse populations, comprehensive restoration-related assessments, and hierarchical statistical approaches are needed to further clarify the relationship between cervical restorations and periodontal health.

## Conclusions

Within the limitations of the present study, cervical restorations were associated with less favorable periodontal parameters compared with non-restored teeth. Restorations with subgingival margins exhibited higher PI, GI, PPD, and CAL values than restorations with supragingival margins. Among the restorative materials evaluated, composite resin restorations were associated with poorer periodontal parameters compared with GIC and RMGIC restorations. These findings suggest that restoration margin location and restorative material may influence periodontal status around cervical restorations. Careful restorative planning, appropriate margin placement, and regular periodontal maintenance may help support periodontal health in restored teeth.
